# Equine-assisted coaching in formerly incarcerated men with histories of substance abuse: a 12-week exploratory study of anger regulation, quality of life, human–horse synchrony, and equine behavior

**DOI:** 10.3389/fpsyg.2026.1832096

**Published:** 2026-05-29

**Authors:** Richard E. Griffioen, Sandra C. Haven-Pross, Hannah Verkuil

**Affiliations:** 1 Department of Applied Research, Aeres University of Applied Sciences, Dronten, Netherlands; 2 Behavioural Science Institute, Radboud University, Nijmegen, Netherlands

**Keywords:** anger regulation, animal-assisted services, equine-assisted coaching, formerly incarcerated individuals, human–horse synchrony, One Health, quality of life, substance use abuse

## Abstract

Equine-assisted interventions are increasingly used as complementary approaches to support psychosocial functioning, yet empirical evidence in justice-involved populations with histories of substance abuse remains limited. The present exploratory quantitative study examined the effects of a 12-week equine-assisted coaching program on anger regulation, quality of life, and human–horse synchrony in formerly incarcerated men, while also monitoring equine behavioral responses from a One Health perspective. Eleven male participants residing in a residential reintegration facility in the Netherlands completed self-report measures at baseline and post-intervention. Anger regulation was assessed using the Anger Bodily Sensations Questionnaire, and quality of life was measured with the PROMIS-29 v2.0 Profile. Human–horse synchrony and equine behavior were analyzed through structured behavioral observation of video-recorded sessions. Group-level analyses showed no statistically significant pre–post differences in anger regulation or quality of life. However, mean anger regulation scores showed a slight increase. Reliable Change Index analyses revealed substantial inter-individual variability: 40% of participants showed reliable increase in score, 20% deterioration in score, and 40% no reliable change. The RCI applied to PROMIS scores indicated decreases in anxiety (64%) and depression (45%), and improvements in social functioning (36%), while other domains showed mixed or stable patterns. Synchrony analyses, based on a smaller subsample, indicated increases in synchrony frequency and duration across the initial sessions, followed by a decline in the final session, possibly reflecting the development of relational attunement. Equine behavior varied significantly across sessions, and negatively coded behaviors occurred more frequently than positively coded behaviors, although these findings should be interpreted contextually rather than as direct indicators of compromised welfare. Overall, the findings suggest that equine-assisted coaching may support emotional awareness, psychosocial functioning, and relational attunement in this population, particularly at the individual level, while underscoring the importance of systematically monitoring horse behavior in animal-assisted services.

## 1 Introduction

Complementary interventions involving animals have been associated with improvements in engagement, coping, and overall well-being across a range of populations (Flynn et al., 2022; Stern and Chur-Hansen, 2019). These populations include individuals with mental health conditions such as depression, anxiety, post-traumatic stress disorder (PTSD), and substance use disorders, as well as individuals with neurodevelopmental conditions such as autism spectrum disorder (Anestis et al., 2014; Lentini and Knox, 2015; O’Haire et al., 2015; Selby and Smith-Osborne, 2013). Within this broader field, equine-assisted services comprise a heterogeneous set of structured, goal-oriented practices in which interaction with horses is integrated into therapeutic, educational, or developmental processes. This heterogeneous field includes for example hippotherapy, equine-assisted therapy (EAT), and equine-assisted coaching (EAC), which differ in their theoretical foundations, level of clinical involvement, and intended outcomes (Wood, 2021; Lentini and Knox, 2015). These approaches are often informed by experiential learning, attachment theory, embodied cognition, and psychophysiological regulation, and existing studies suggest that they may support emotional regulation, self-efficacy, social functioning, and stress reduction (Stern and Chur-Hansen, 2019). Hippotherapy, for example, is typically physiotherapy-based and focuses on motor functioning, whereas EAT involves licensed mental health professionals targeting psychological outcomes; in contrast, EAC is generally non-clinical and emphasizes experiential learning and self-reflection through interaction with the horse (Debuse et al., 2005; Selby and Smith-Osborne, 2013; Wood, 2021).

Equine-assisted coaching represents a non-clinical, growth-oriented branch of equine-assisted services that focuses primarily on self-awareness, leadership, communication, and interpersonal functioning rather than on the formal treatment of psychiatric disorders. In this context, horses serve as interactive partners in experiential exercises that invite reflection on behavior, boundaries, and relational dynamics. Despite increasing practical use, rigorous empirical evidence remains limited, particularly for highly vulnerable populations (Kendall et al., 2015; Fine and Andersen, 2021).

The present study focused on formerly incarcerated men with histories of substance abuse. Substance dependence is a chronic and relapsing condition characterized by compulsive use, impaired control, tolerance, withdrawal symptoms, and continued use despite harmful consequences (Liu and Li, 2018; Koob and Volkow, 2016). Individuals with histories of substance abuse commonly face difficulties in emotion regulation, emotional awareness, and social functioning, complicating reintegration following incarceration (Koob and Volkow, 2016). Whereas conventional evidence-based approaches remain essential, complementary interventions may benefit those who struggle to engage with predominantly verbal forms of treatment.

Treatment is further complicated by high drop-out rates, a meta-analysis reported an average of 30.4% in psychosocial interventions (Lappan et al., 2020) with similar rates in dual-diagnosis populations (Bouchard et al., 2022), suggesting a meaningful subgroup is not adequately reached. Complementary experiential approaches may strengthen engagement and support emotional regulation. Among these, equine-assisted interventions show preliminary evidence of improving treatment involvement and psychosocial functioning, though findings remain limited by methodological constraints (Diaz et al., 2022; Machová et al., 2023). These findings suggest equine-assisted coaching may be valuable as an intervention for individuals with low motivation, prior treatment dropout, or complex psychosocial need.

Evidence for equine-assisted interventions in forensic and high-risk populations is promising but inconsistent. Prison-based programs have been associated with reduced recidivism risk and improved relational and emotional competencies, though results vary across outcome measures (Bachi, 2012). Studies with at-risk youth, while limited by small sample sizes (e.g., *n* = 7), report improvements in self-confidence, emotional regulation, and interpersonal functioning (Burgon, 2011). Controlled research with trauma-exposed youth (*n* = 68) shows possible reductions in post-traumatic stress symptoms, though these effects may not surpass those observed with standard treatment (Mueller and McCullough, 2017).

Building on these findings, animal-assisted interventions may be especially relevant in correctional and post-correctional contexts. Prior work suggests that these interventions can support psychosocial well-being, skill development, and adaptive behavior among incarcerated individuals (*n* = 17) (Kunz-Lomelin and Nordberg, 2020). In a Canadian prison context, therapy dogs were perceived as sources of love and support, and the resulting human–animal bond appeared to foster emotional awareness and improved conduct (*n* = 6) (Dell and Poole, 2015). These findings suggest that animal-assisted approaches may create a nonverbal and relational pathway to emotional engagement, one that equine-specific programs may be particularly well placed to offer.

Despite this promise, the existing literature on animal-assisted interventions in incarcerated populations remains limited. Most studies use relatively small sample sizes (e.g., *n* = 3–24; Contalbrigo et al., 2017; Eaton-Stull et al., 2020; 2025; Dell et al., 2019; Smith et al., 2023) and should be considered preliminary. Research on equine- assisted interventions in these settings is even less established, generally involving small samples (e.g., *n* = 5–12) and relying on qualitative or mixed-methods designs (Arditti et al., 2020; Robinson-Edwards et al., 2019; Thody, 2025).

Beyond correctional settings, animal-assisted interventions have also been applied to populations with substance use disorders, with research showing potential benefits such as improved treatment engagement, self-efficacy, and emotional well-being, although findings remain tentative due to similar methodological constraints (Diaz et al., 2022). Similarly, a clinical study reported improvements in mood, quality of life, and social functioning following equine assisted interventions (Machová et al., 2023). Together, these data suggest that animal-assisted approaches may support recovery processes across a range of clinical and psychosocial populations, calling for further investigation in post-correctional populations with histories of substance abuse.

Several theoretical perspectives support this assumption. The biophilia hypothesis proposes that humans have an innate tendency to seek connection with other living beings, making animals potentially important sources of comfort and emotional engagement (Wilson, 1984). Human–animal interaction has also been associated with psychophysiological regulation, including oxytocin release and reductions in arousal (Beetz et al., 2012). In addition, the human–animal bond may provide a non-judgmental relational context in which attachment-related processes and social support can emerge (Enders-Slegers, 2000; Kruger and Serpell, 2010).

A particularly relevant concept in this context is behavioral synchrony, defined as the temporal coordination of actions, movements, or affective expressions between interaction partners. Synchrony has been linked to bonding, cooperation, and communication and has been documented in both human–human and human–animal interactions (Griffioen et al., 2019, 2020; Maeda et al., 2021). Because synchrony requires attunement, reciprocal adjustment, and sensitivity to context, it may represent one mechanism through which equine-assisted interventions exert their effects (van der Steen et al., 2019).

The present study was further informed by a One Health perspective, which emphasizes the interdependence of human, animal, and environmental well-being (Hediger et al., 2019; Jegatheesan et al., 2018). From this perspective, animal-assisted services should be evaluated not only in terms of participant outcomes but also in relation to the welfare and behavioral responses of the animals involved. Evidence suggests that horses experience a range of affective states during equine-assisted services (EAS) (Visser et al., 2025).

Therefore, welfare assessments should include indicators of both positive and negative behavior, as contemporary frameworks emphasize that good welfare requires not only the minimization of negative states such as pain, fear and stress, but also the promotion of positive experiences and affective states (Mellor et al., 2020). Systematic monitoring of equine behavior is therefore essential to ensure ethically responsible practice and to strengthen the empirical foundation of equine-assisted interventions.

However, important gaps remain. Existing studies often rely on small samples, focus primarily on human outcomes, and rarely integrate systematic assessment of equine welfare alongside participant functioning. In addition, little is known about how individuals with difficulties in emotion regulation and social functioning, such as those with histories of substance abuse, interact with horses in coaching contexts and how this may relate to both human outcomes and equine responses.

The aim of this study was to examine the effects of a 12-week equine-assisted coaching program on anger regulation, quality of life, and human–horse synchrony among formerly incarcerated men with histories of substance abuse, while also monitoring equine behavior during the sessions.

## 2 Materials and methods

### 2.1 Study design

A quantitative exploratory design was utilized, integrating standardized self-report questionnaires with structured behavioral observations. The study examined the effects of a 12-week equine-assisted coaching intervention on anger regulation, quality of life, human–horse synchrony, and equine behavioral responses among formerly incarcerated men with histories of substance abuse. Changes in anger regulation and quality of life were measured from baseline to post-intervention, while human–horse synchrony was assessed across four intervention sessions. Equine behavioral responses were also systematically observed throughout the sessions.

### 2.2 Participants and setting

The study population consisted of 11 male clients participating in a rehabilitation program jointly organized by Terwille Addiction Care and DeVlugtCoaching. All participants had a judicial background and had previously been incarcerated for drug-related offenses. Questionnaire data were collected for all 11 participants.

During the study period (September 2022–March 2023), participants resided at De Spetse Hoeve, a Terwille facility in Veelerveen, the Netherlands. They were either recently released or conditionally released from prison and were enrolled in a structured reintegration and recovery program of approximately 6 months. The program included multiple weekly therapeutic activities and one equine-assisted intervention session per week. Participants ranged in age from 20 to 55 years.

Because clients were at different stages of their rehabilitation trajectories, participation in the study was aligned with their treatment schedules to avoid postponing therapy for research purposes.

### 2.3 Ethics

This study involved the monitoring of an existing program and did not introduce any additional interventions beyond routine practice; therefore, ethical approval was not required according to institutional guidelines. All participants provided written informed consent prior to participation and were informed that they could withdraw from the study at any time without consequences.

Ethical approval was not required for the animal component of this study because no invasive procedures or experimental manipulations involving animals were conducted.

### 2.4 Horses and intervention context

The equine participants consisted of four Konik horses (three mares and one gelding). The horses were accustomed to human interaction but were not ridden, allowing their natural behavior to remain largely intact. They were housed outdoors in pasture environments with continuous access to water, forage, and shelter. Their ages ranged from 2 to 5 years.

The equine-assisted coaching intervention consisted of structured, one-on-one sessions involving the participant, a trained facilitator, and a horse. Each session (~30 min) followed a general framework, while allowing for adaptation to individual needs and responses. Sessions typically began with a brief check-in, followed by experiential activities with the horse, and concluded with a guided reflection.

Activities primarily involved groundwork (i.e., interactions with the horse from the ground rather than riding) and included tasks such as leading the horse through obstacles (leadership tasks), directing movement without physical force, and engaging in proximity-based exercises aimed at increasing awareness of body language and emotional states. Relaxation-based exercises were also incorporated, focusing on breathing, posture, and attunement to the horse’s responses.

The intervention was grounded in principles of experiential learning and non-verbal communication. Participants were encouraged to reflect on their interactions with the horse, with particular attention to themes such as emotional regulation, boundary setting, and interpersonal dynamics. The horse’s behavior was used as a source of immediate, non-verbal feedback, facilitating insight into participants’ behavioral patterns.

Although sessions were individualized, the overall structure and therapeutic focus were consistent across participants, ensuring a comparable intervention framework.

### 2.5 Recording procedure

**Figure 1:**
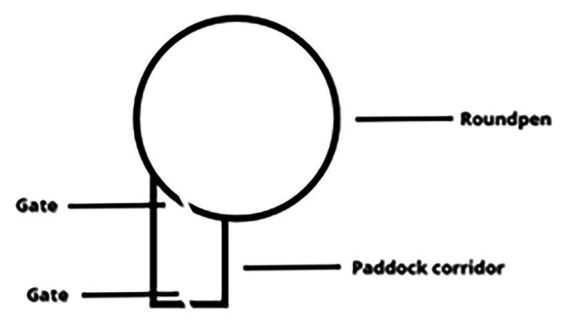
Round pen.

All intervention sessions were video recorded using two Sony HD CX410 cameras. The sessions took place in a fenced round pen (see Figure 1). The remaining herd stayed visible in an adjacent pasture, thereby preserving visual social contact for the focal horse. One camera was mounted on a tripod and manually tracked the movements of the participant. The other was mounted on a PIXIO v2.8.1 system (Pixio Move 'N See, Vannes, France). This PIXIO tracked a device attached to the horse via a band positioned behind the withers.

### 2.6 Measures

#### 2.6.1 Anger regulation

Anger regulation was assessed using the Anger Bodily Sensations Questionnaire (ABSQ), a validated self-report instrument assessing bodily sensations associated with anger in interpersonal contexts (Zwets et al., 2014). The ABSQ contains nine items rated on a 5-point Likert scale. Higher scores indicate greater awareness of bodily activation in anger-provoking situations.

#### 2.6.2 Quality of life

Quality of life was assessed using the PROMIS-29 v2.0 Profile (Hanmer et al., 2020). This instrument measures physical functioning, anxiety, fatigue, sleep disturbance, depression, social participation, and pain-related domains. It was selected because of its strong psychometric properties and sensitivity to change (Elsman et al., 2022; Hinchcliff et al., 2011; Selewski et al., 2013). The PROMIS-29 Profile reports on health status using T-scores (mean = 50, SD = 10). Higher scores indicate more of the measured concept, meaning worse symptoms for domains like anxiety or pain, but better functioning for physical and social domains. Interpretation of the scores is domain dependent. For physical functioning and participation in social roles, higher T-scores indicate better functioning. In contrast, for anxiety, depression, fatigue, and sleep disturbance, higher T-scores reflect more severe symptoms. Accordingly, changes in T-scores over time (from baseline to week 12) were interpreted in line with this directionality: increases in functioning domains represent improvement, whereas decreases indicate deterioration; for symptom domains, decreases represent improvement and increases indicate worsening symptoms.

#### 2.6.3 Human–horse synchrony

Human-horse synchrony was assessed through structured behavioral observation using a predefined ethogram and observation protocol. Video data were analyzed using Mediacoder software (Bos and Steenbeek, 2010). Synchrony measures focused on coordinated or mirrored patterns of movement and posture between participant and horse. These observations were guided by the protocol and ethogram. The behaviors that were coded and analyzed for participants included: walking direction toward the horse, walking direction toward a goal, moving forward, and stopping movement. For the horses, the coded behaviors included: walking direction toward the client, walking direction toward a goal, moving forward, and stopping movement. These behavioral categories were based on the synchronization framework described by Griffioen et al. (2020). The ethogram is available via Supplementary Table S1. For the synchrony analyses, only the data from seven participants could be included.

#### 2.6.4 Equine behavior

A total of 21 distinct equine behaviors were video recorded across 14 sessions. These behaviors were adopted from the study on equine behavioral responses in Equine Assisted Services by Visser et al. (2025) and represent behaviors indicative of both positive and negative mental states. The ethogram can be found in the Supplementary Table S2. Behavioral data were coded using BORIS (Behavioral Observation Research Interactive Software; version 8.x) (Friard and Gamba, 2016). For each session, the frequency of each behavior was quantified based on analyses of video recordings. Behaviors were subsequently categorized as positive or negative; the categorization was adapted from Visser et al. (2025), after which total category frequencies per session were calculated. A comprehensive overview of positive and negative behaviors is available in the Supplementary material.

### 2.7 Procedure

The intervention period lasted 12 weeks. Video recordings were obtained at four time points: weeks 1, 4, 8, and 12. The ABSQ and PROMIS-29 questionnaires were administered at week 1 and week 12.

### 2.8 Statistical analysis

Differences in ABSQ and PROMIS-29 scores between baseline and post-intervention were analyzed using Wilcoxon signed-rank tests to assess within-group (pre-post) changes. In addition, the Reliable Change Index (RCI) was calculated using a reliability coefficient of *r* = 0.90. The standard error of measurement (SEM) and standard error of difference (Sdiff) were computed using this reliability estimate. An RCI value exceeding ±1.96 was considered indicative of statistically reliable change at the 95% confidence level. Because the equine behavior data consisted of repeated non-normally distributed measurements, a Friedman test was used to examine differences in the frequency of the 21 observed behaviors across the 14 sessions. *Post hoc* Conover tests were performed to identify pairwise differences between behaviors. To compare the total frequency of positive and negative equine behaviors within sessions, a paired Wilcoxon signed-rank test was conducted. Statistical analyses were conducted using Microsoft Excel 365 (Microsoft Corporation, Redmond, WA, United States), IBM SPSS Statistics version 28.0 (IBM Corporation, Armonk, NY, United States), and RStudio 2024.12.0 + 467 (R Foundation for Statistical Computing, Vienna, Austria). A *p*-value of less than 0.05 was used as the threshold for statistical significance.

## 3 Results

### 3.1 Anger regulation

Mean ABSQ scores showed a small increase from pre-intervention (*M* = 32.80, SD = 7.97) to post-intervention (*M* = 34.70, SD = 9.46), suggesting a slight overall increase in symptom severity at the group level. The Wilcoxon signed-rank test showed no statistically significant difference between baseline and post-intervention scores at the group level, *p* = 0.6818.

**Figure 2:**
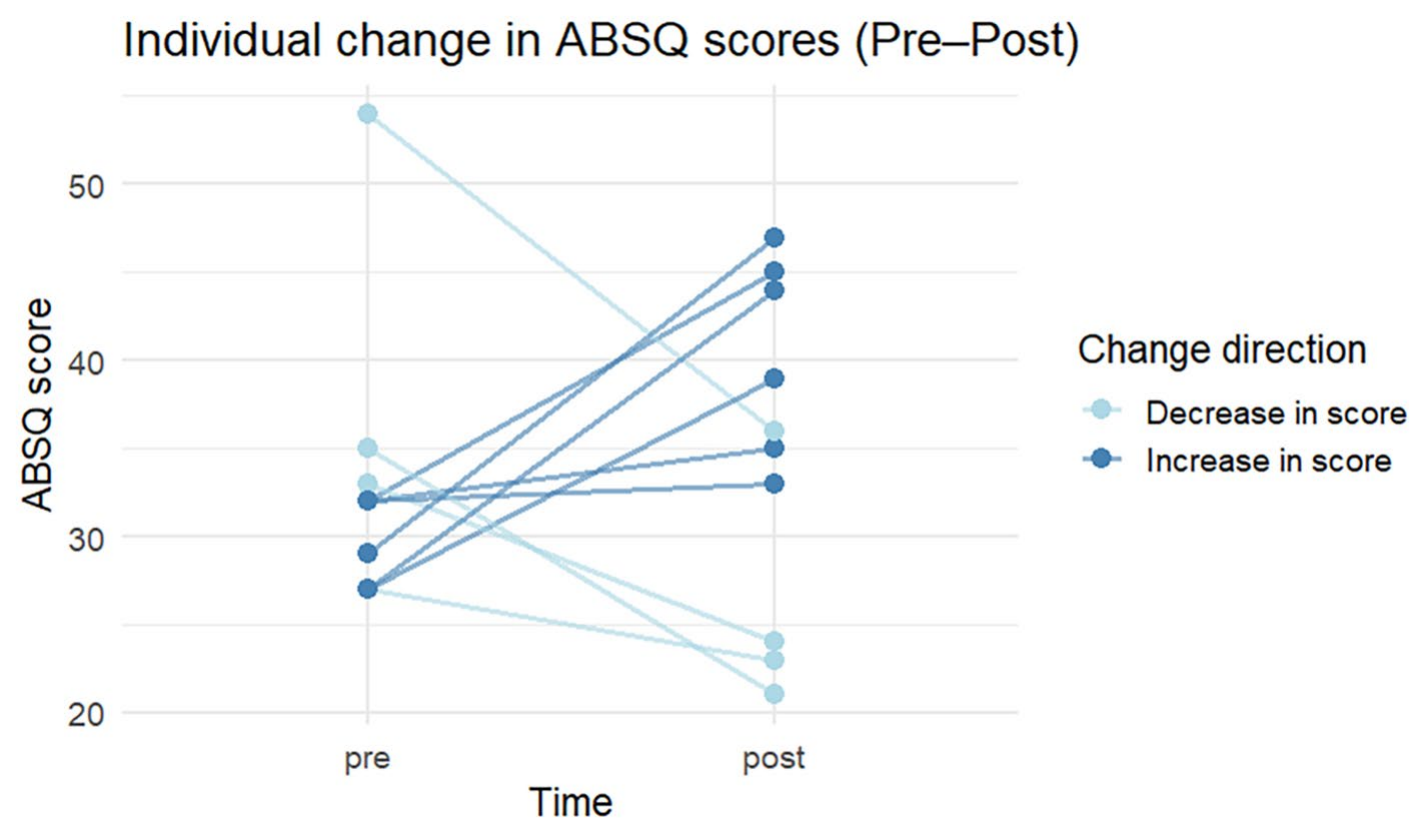
Spaghetti plot showing individual changes in ABSQ scores from pre- to post-intervention. Each line represents an individual participant, with scores plotted at baseline and follow-up. Line colors indicate reliable change index (RCI) classification, representing a decrease in scores (dark blue), an increase in scores (light blue), or no reliable change (gray).

Reliable Change Index showed that at individual level, 4 participants (40%) demonstrated reliable improvement (i.e., increased scores; P2, P7, P8, P10), 2 participants (20%) showed reliable deterioration (i.e., decreased scores; P6, P11), and 4 participants (40%) showed no reliable change (P1, P3, P4, P5) (Figure 2).

### 3.2 Quality of life

Quality of life was assessed using the PROMIS-29. Domain-level comparisons between baseline and week 12 showed no statistically significant group-level differences across physical functioning, anxiety, fatigue, sleep disturbance, depression, or ability to participate in social roles and activities. No inferential tests were conducted for pain interference or pain intensity. Pain interference showed no change across participants, and pain intensity varied minimally.

The results are summarized in Table 1. Interpretation of the scores is domain dependent. For physical functioning and participation in social roles, higher T-scores indicate better functioning. In contrast, for anxiety, depression, fatigue, and sleep disturbance, higher T-scores reflect more severe symptoms. Accordingly, changes in T-scores over time (from baseline to week 12) were interpreted in line with this directionality: increases in functioning domains represent improvement, whereas decreases indicate deterioration; for symptom domains, decreases represent improvement and increases indicate worsening symptoms.

**Table 1:** PROMIS-29 domain-level comparisons between baseline and week 12.

Domain	*p*-value
Physical functioning	0.590
Anxiety	0.099
Fatigue	0.080
Sleep disturbance	0.933
Depression	0.406
Ability to participate in social roles/activities	0.141

**Figure 3:**
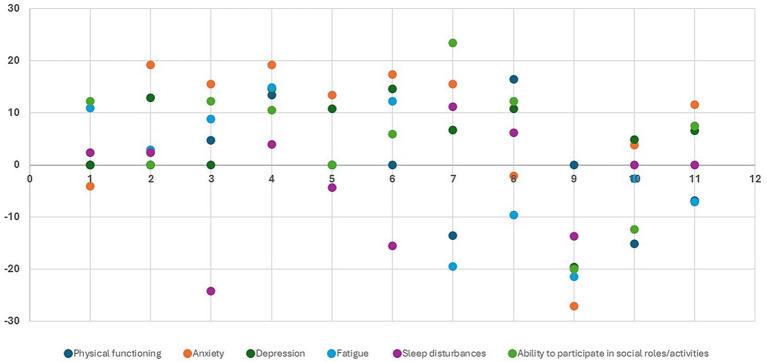
Scatterplot showing individual PROMIS-29 T-score changes from baseline to week 12 across domains. Each number (1-11) represents an individual participant, the colors represent the 6 domains of the PROMIS-29: physical functioning (dark blue), anxiety (orange), depression (dark green), fatigue (turquoise), sleep disturbances (purple), ability to participate in social roles/activities (light green). Positive values indicate better functioning or fewer symptoms, whereas negative values reflect poorer functioning or more severe symptoms, depending on the domain. The spread of the dots illustrates variability across individuals or measurements.

Although group-level differences were not statistically significant, individual-level patterns suggested potentially meaningful improvements in anxiety, fatigue, sleep disturbance, and social participation for several participants. Domain-specific individual trajectories are presented in Figure 3.

**Figure 4:**
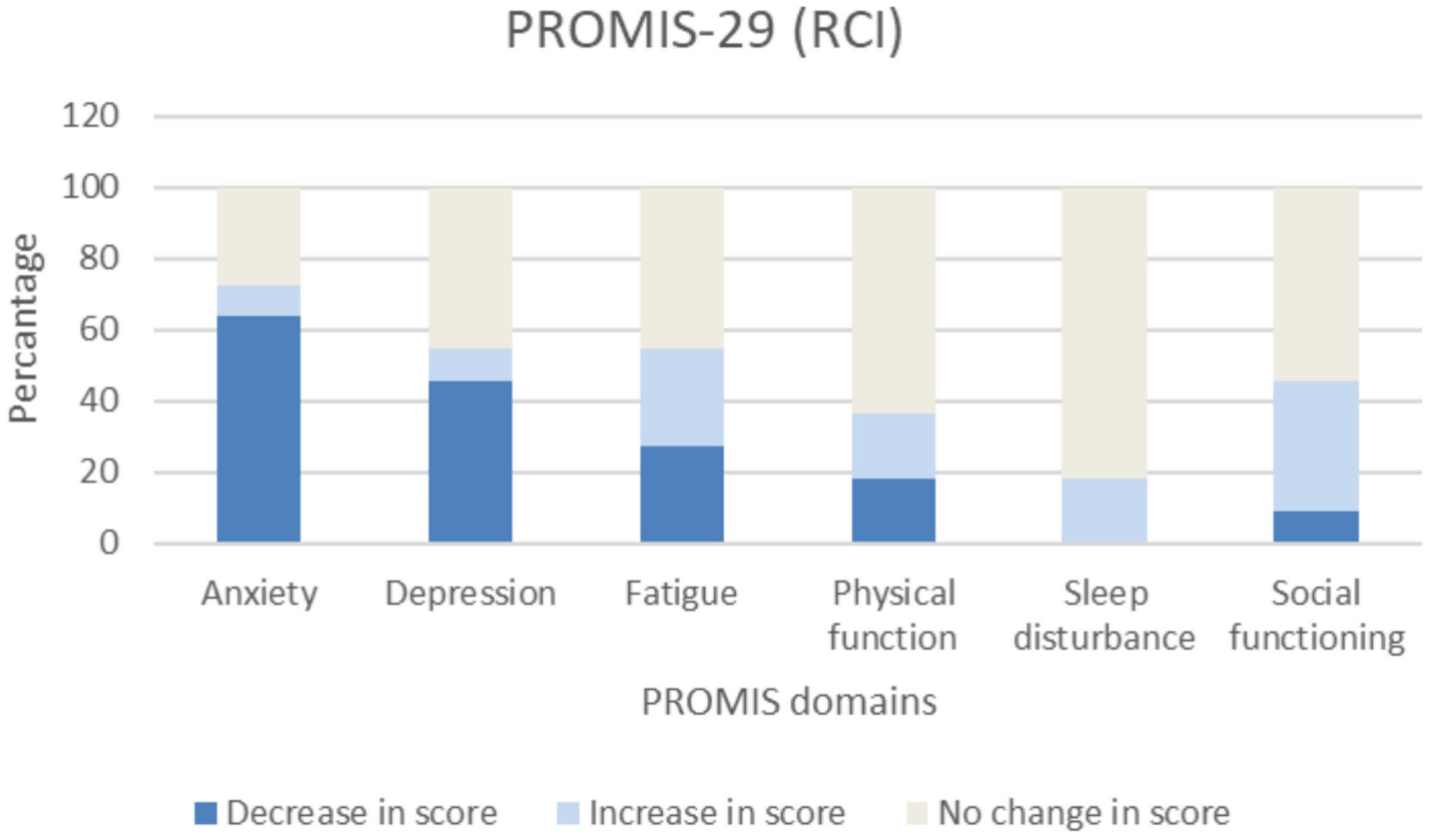
Bar chart showing individual Reliable Change Index (RCI) classifications from week 1 to week 12 across domains. The *y*-axis indicates the percentage of participants, and the *x*-axis represents the six outcome domains: physical functioning, anxiety, depression, fatigue, sleep disturbance, and social functioning. Cell colors reflect RCI-based change classifications, indicating reliable decrease in scores (light blue), reliable increase in scores (blue), and no reliable change (gray), representing non-significant change within measurement error.

The Reliable Change Index (RCI) results for the PROMIS-29 domains were as follows. For physical functioning, 2 participants showed a decrease in scores, 2 showed an increase, and 7 showed no reliable change. For anxiety, 7 participants showed a decrease, 1 showed an increase, and 3 showed no reliable change. For depression, 5 participants showed a decrease, 1 showed an increase, and 5 showed no reliable change. For fatigue, 3 participants showed an increase, 3 showed a decrease, and 5 showed no reliable change. For sleep disturbance, 2 participants showed an increase, and 9 showed no reliable change. For social functioning, 4 participants showed an increase, 1 showed a decrease, and 6 showed no reliable change. An overview of these individual-level changes is presented in Figure 4.

### 3.3 Human–horse synchrony

For the synchrony analyses, only the data from seven participants could be included. Several participants completed sessions only with the use of a rope, which did not allow adequate evaluation of synchrony in posture and movement between participants and horse.

#### 3.3.1 Synchrony frequency

**Figure 5:**
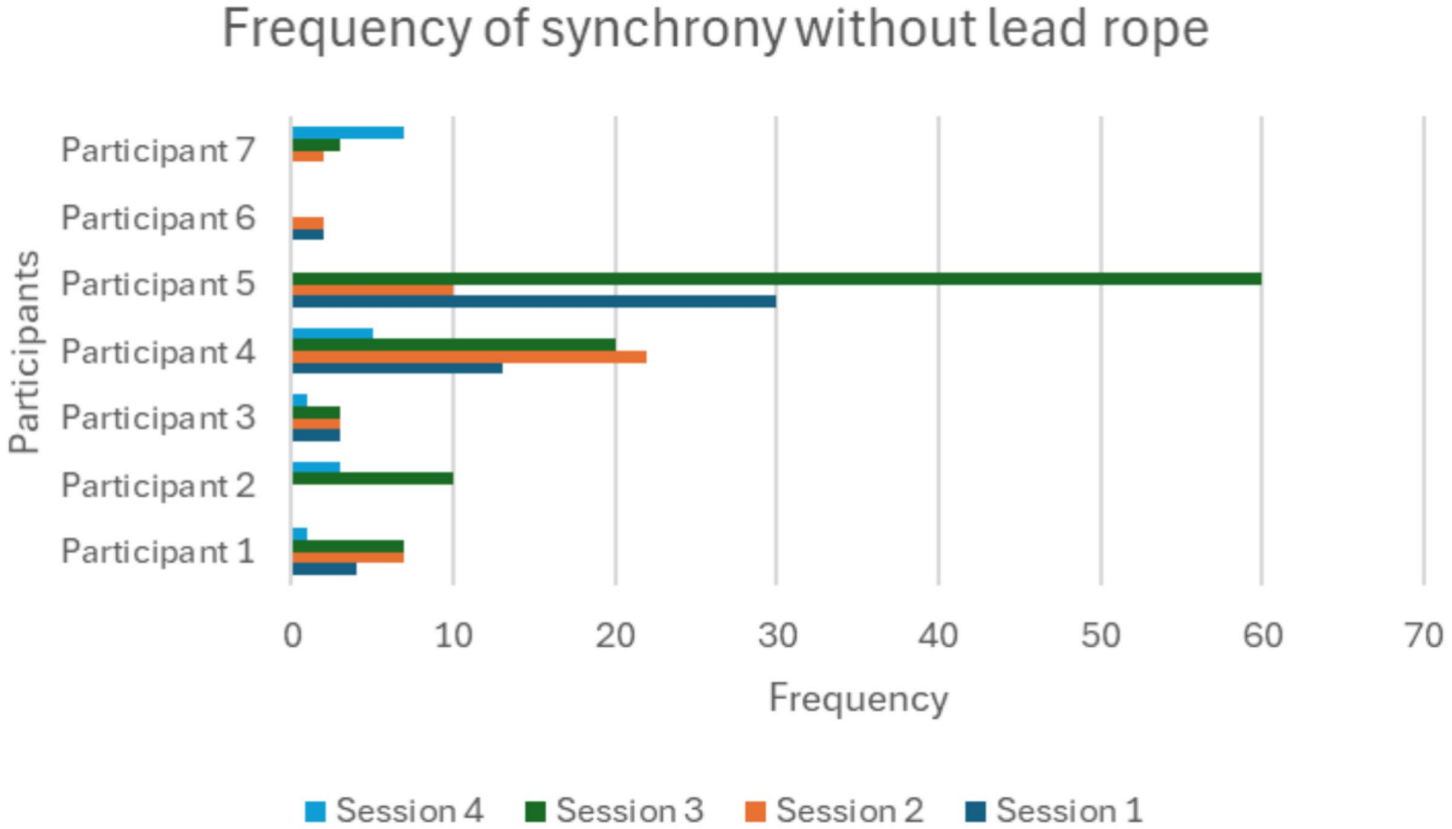
Frequency of human–horse synchrony across four sessions without the use of a lead rope. The bar colors represent Session 1 (blue), Session 2 (orange), Session 3 (green), and Session 4 (turquoise). The *x*-axis represents frequency per session of 30 minutes, and the *y*-axis represents the sessions per participant. The longer the bar indicates a higher amount of time the horse and participant were synchronizing.

Synchrony frequency generally increased across the initial sessions for most participants, as shown in Figure 5. Participants 1 and 2 showed a gradual rise toward later sessions, while Participant 3 remained relatively stable early on. Participant 4 increased initially but then declined. Participant 5 consistently showed higher values and continued to increase. Participant 6 remained stable across two sessions, whereas Participant 7 showed an increase. Among the first four participants, a decrease appeared in the final session. This reduction may reflect contextual changes or environmental novelty (e.g., the introduction of a ball game) rather than a true decline in attunement.

#### 3.3.2 Synchrony duration

**Figure 6:**
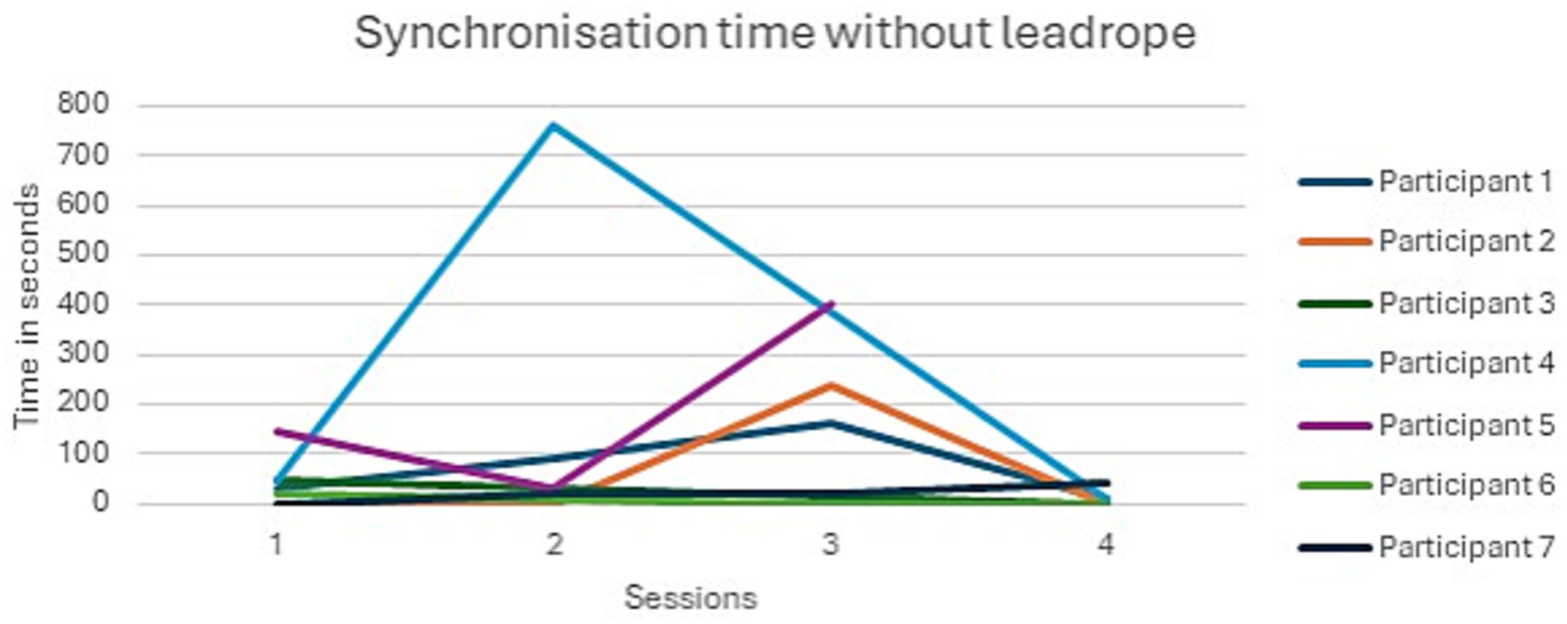
Duration of human–horse synchrony across four sessions without the use of a lead rope. The lines represent Participant 1 (blue), Participant 2 (orange), Participant 3 (dark green), Participant 4 (turquoise), Participant 5 (purple), Participant 6 (light green), and Participant 7 (dark blue). The *y*-axis represents time in seconds, and the *x*-axis represents the sessions in chronological order. The lines show how long the participants and the horses were synchronized.

A similar pattern was observed for synchrony duration. For three of the seven participants, duration increased across the initial sessions and declined in the final session. One participant showed a sharp increase in Session 2, followed by a decrease thereafter. Participant 5 decreased in Session 2 but increased again in Session 3. As with frequency, Participants 6 and 7 exhibited lower values overall. Session-level synchrony duration data are presented in Figure 6.

### 3.4 Equine behavioral responses

Equine behavior was evaluated by a blinded observer who had no prior knowledge of the horse assigned to each session, thereby minimizing potential observer bias. No clear indicators of compromised welfare were identified. The horses remained within a normal behavioral range throughout the study period. In line with the study protocol and established coaching practices, the coach continuously monitored for behavioral signs of excessive discomfort and was required to terminate the session should such signs be observed (Ekholm Fry, 2021). No sessions were terminated on these grounds.

The Friedman test revealed a statistically significant effect of behavior type across the 14 sessions, χ^2^(20) = 234.50, *p* < 0.001, Kendall’s *W* = 0.84, indicating substantial variation in the frequency with which specific equine behaviors occurred.

*Post hoc* Conover tests showed that behaviors such as tail swishing, ears forward, head held high, and head held low occurred significantly more frequently than rarely observed behaviors, including loose tail posture, rolling, snorting, blowing, and self-grooming.

The paired Wilcoxon signed-rank test comparing positive and negative behavior categories showed a significant difference (*W* = 102.00, *z* = 3.11, *p* < 0.001). Negatively coded behaviors (e.g., ears flattened and tail swishing) occurred more frequently than positively coded behaviors (e.g., ears forward and head held low) across sessions. Descriptive statistics indicated higher mean frequencies for negative behaviors (*M* = 154.14, SD = 71.60) than for positive behaviors (*M* = 97.29, SD = 43.54).

## 4 Discussion and conclusion

The present study explored the effects of a 12-week equine-assisted coaching intervention in formerly incarcerated men with histories of substance abuse, focusing on anger regulation, quality of life, human–horse synchrony, and equine behavioral responses. Given the small sample size and exploratory design, the findings should be interpreted with caution. Nevertheless, several patterns merit discussion.

No statistically significant group-level improvement in anger regulation was observed; however, a slight overall increase in mean group scores was found. At the individual level, 40% of participants showed an increase in scores. This finding aligns with qualitative reports from several participants indicating increased awareness of anger-related bodily sensations over time. Rather than indicating deterioration, this pattern may reflect greater emotional and bodily awareness. Substance use has been associated with emotional numbing and reduced access to internal states (Koob and Volkow, 2016), whereas work with horses may require emotional congruence and embodied awareness (Brandt, 2013; McCormick, 1997). In this sense, higher ABSQ scores may signal a clinically meaningful increase in self-awareness rather than poorer regulation.

Likewise, no statistically significant group-level changes in quality of life emerged, but individual statistics suggested improvement in anxiety, depression, and participation in social roles. This pattern is broadly consistent with earlier studies indicating beneficial effects of equine-assisted interventions on anxiety, mood, and psychosocial functioning (Earles et al., 2015; Jeon and Son, 2021; O’Haire et al., 2015). Improved participation in social roles may be related to the non-verbal, relational nature of the work, which requires clear communication, awareness of boundaries, and behavioral congruence (Porter-Wenzlaff, 2007; Rothe et al., 2005; Veenstra et al., 2017).

Although no statistically significant changes were observed at the group level for the PROMIS-29 and ABSQ scores, several individuals showed meaningful changes. In patient-reported outcome research, group means may mask heterogeneity in individual responses. Therefore, within-person change and responder analyses are often considered informative complements to group comparisons (Revicki et al., 2008). Moreover, differences that appear small at the group level may be clinically relevant at the individual level (Elsman et al., 2022).

The synchrony findings tentatively suggest that human–horse attunement increased during the initial phase of the intervention. Both synchrony frequency and duration generally rose across the first sessions for most participants, which may reflect gradual improvements in non-verbal communication, relational attunement, and emotional regulation. This trajectory is consistent with theoretical accounts that position synchrony as a potential mechanism of therapeutic change in animal-assisted interventions (Griffioen et al., 2019, 2020; van der Steen et al., 2019). The decline observed in the final session may have resulted from changes in session context or novelty in the environment rather than by a genuine loss of attunement.

This issue is particularly relevant for individuals with histories of substance abuse, who frequently experience challenges in emotion regulation, emotional awareness, and social functioning (Koob and Volkow, 2016; Sinha, 2008). These challenges may influence the consistency and predictability of human–horse interactions during equine-assisted coaching. Horses are highly sensitive to human cues, including posture, facial expressions, and body odors (Merkies and Franzin, 2021; Scopa et al., 2019), so dysregulated emotional states in clients could plausibly elicit stress responses in the animals. Recent studies have shown that certain equine-assisted activities, such as grooming, can trigger stress-related responses in horses (Rudd et al., 2024), while humans often fail to consistently recognize horses’ affective states (Müller-Klein et al., 2024). Despite this bidirectional dynamic, current evidence does not yet demonstrate that dysregulated human emotional signals consistently impair equine welfare; representing a significant gap that warrants further investigation (Kelly et al., 2021; Merkies and Franzin, 2021). Collectively, these findings highlight the importance of cautious interpretation of equine behavioral responses and the need for systematic monitoring of welfare in equine-assisted services contexts.

The equine behavior findings showed substantial variability across sessions. Although the higher frequency of negatively coded behaviors compared to positively coded ones could suggest that horses experienced discomfort or stress during the intervention, this interpretation requires important qualification. The ethogram included a greater number of negatively coded behaviors, structurally inflating their occurrence. Additionally, *post hoc* Conover test demonstrated that both negatively and positively coded behaviors were among the most frequently observed, with tail swishing and head held high (negative) appearing alongside ears forward and head held low (positive). These observations indicate a more nuanced pattern of behavioral expression rather than a uniformly negative welfare state. In structured intervention settings, behaviors categorized as negative may also reflect alertness, environmental responsiveness, or task-related engagement rather than distress. This interpretive complexity is compounded by the nature of equine behavioral signs: while some have a relatively clear valence (e.g., ears forward versus ears flattened), many are context-dependent and only gain meaning when interpreted in combination with other behavioral cues (Lesimple, 2020). Welfare assessment in horses therefore requires a multimodal and context-sensitive approach, as single indicators may not reliably reflect underlying affective states (De Santis et al., 2017). Nevertheless, the repeated occurrence of certain behaviors across sessions should not be disregarded and may still indicate welfare challenges that merit attention.

The relatively higher occurrence of negatively coded behaviors in the present study contrasts with findings reported by Visser et al. (2025), who observed a greater occurrence of behaviors with positive valence during human–horse interactions. Such differences may reflect variations in study context, intervention structure, or behavioral coding approaches. A key difference between the two studies is the participant population: the present study involved only individuals with histories of substance abuse, who may have presented with greater emotional dysregulation than the more diverse population studied by Visser et al. (2025), potentially contributing to more stress-related equine responses. From a One Health perspective, these findings highlight the importance of interpreting equine behavioral responses contextually and as part of broader behavioral patterns rather than as isolated indicators of welfare (Hediger et al., 2019; Jegatheesan et al., 2018).

Several limitations should be acknowledged. First, the sample was small and variable, with participants at different stages of rehabilitation. Second, synchrony analyses were conducted on a reduced subsample. Third, categorizing equine behaviors as positive or negative likely oversimplified the complexity of animal behavior. In both the study design and routine equine-assisted services practice, sessions are carefully monitored by the coach, and it is standard procedure to terminate a session if a horse shows signs of excessive discomfort. No such interventions were necessary during the study. However, the small sample size (*n* = 4) restricts the ability to draw conclusions regarding individual variability in equine welfare responses, and subtle or transient differences between horses may not have been fully detected. Future studies with larger samples and designs specifically powered to detect inter-individual variation are needed to clarify how horses experience equine-assisted coaching contexts. Fourth, the study relied primarily on behavioral data and did not incorporate physiological indicators of equine stress or well-being. Fifth, the study did not control for concurrent treatments. Participants were involved in ongoing care trajectories, and additional interventions were neither standardized nor systematically assessed, limiting the attribution of the observed effects specifically to the equine-assisted intervention (McLellan et al., 2000). The observed variability in outcomes, ranging from clear improvements to minimal change, may partly reflect differences in concurrent care and treatment exposure (Brorson et al., 2013). Notably, participants showing the greatest improvements in ABSQ and PROMIS-29 scores tended to have more pronounced baseline difficulties in emotional regulation and quality of life. This may suggest differential responsiveness, where individuals with greater affective or interpersonal impairments benefit more from experiential, non-verbal interventions. Future research should more systematically assess concurrent treatments and identify predictors of response to better delineate target populations (Kazdin, 2007; Lutz et al., 2019).

Despite these limitations, this study contributes to the emerging literature on equine-assisted coaching in justice-involved populations with histories of substance abuse. The findings suggest potential benefits for emotional awareness, psychosocial functioning, and relational attunement, while also highlighting the importance of systematic monitoring of horse behavior in intervention settings. Future research should use larger and more stable samples, standardized session structures, longer follow-up periods, and multimodal welfare assessments that combine behavioral and physiological indicators.

## Data Availability

The raw data supporting the conclusions of this article will be made available by the authors on reasonable request.
