# Supplementary material for: Equine-assisted coaching in formerly incarcerated men with histories of substance abuse: a 12-week exploratory study of anger regulation, quality of life, human–horse synchrony, and equine behavior
Source: Front Psychol. 2026 May 29;17:1832096. doi: 10.3389/fpsyg.2026.1832096 (PMC13261911; doi:10.3389/fpsyg.2026.1832096)
Supplement: Supplementary file 1 [file table_1.docx]

Table S1

Supplementary material_Ethogram synchrony

| **Client behaviour** | **Description** | **Code** | **Source** |
| --- | --- | --- | --- |
| Stop moving | The client stops moving in a specific direction. | 0 | Griffioen, van der Steen, Verheggen & Enders-Slegers (2020) |
| Walking direction toward horse | The client takes one or more clear steps toward the horse. The horse does not need to be reached. | 1 | Griffioen, van der Steen, Verheggen & Enders-Slegers (2020) |
| Walking direction toward target | The client takes clear steps toward a goal such as the coach or objects (e.g., cones or an obstacle). The object does not need to be reached. | 2 | Griffioen, van der Steen, Verheggen & Enders-Slegers (2020) |
| Walking forward | The client takes clear steps in the round pen without a specific target, such as an object, coach, or horse. This also applies when the coach instructs the client to walk around. | 3 | Griffioen, van der Steen, Verheggen & Enders-Slegers (2020) |
| Walking forward with rope | The client takes clear steps in the round pen while the horse is attached to a lead rope. This also applies when the client attempts but is unable to move the horse, even if standing still. Includes completing a course with the horse. | 4 | — |
| Out of frame | The client moves out of view. | 5 | — |

| **Walking direction** | | | |
| --- | --- | --- | --- |
| **Client** | | **Horse** | |
| Behaviour code | Time in seconds | Behaviour code | Time in seconds |
|  |  |  |  |
