# Supplementary material for: Equine-assisted coaching in formerly incarcerated men with histories of substance abuse: a 12-week exploratory study of anger regulation, quality of life, human–horse synchrony, and equine behavior
Source: Front Psychol. 2026 May 29;17:1832096. doi: 10.3389/fpsyg.2026.1832096 (PMC13261911; doi:10.3389/fpsyg.2026.1832096)
Supplement: Supplementary file 2 [file table_2.docx]

| Behavior label | Definition used in the study | Associated valence |
| --- | --- | --- |
| Ears forward | Both ears are held simultaneously upright and directed forward for at least 3 seconds. | Positive |
| Ears flattened | Both ears are simultaneously directed downward and backward, forming an angle of less than 45 degrees with the neck. | Negative |
| Ears active | The horse's ears move independently, shifting from front to back for at least 3 seconds, with the movement speed potentially varying between the left and right ear. | Positive |
| Head high | The nose or mouth is above an imaginary horizontal line drawn from the lowest point of the horse's back and stays in this position for at least 3 seconds. | Negative |
| Head low | The nose or mouth is below an imaginary horizontal line drawn from the lowest point of the chest and remains in this position for at least 3 seconds. | Positive |
| Head shaking | The head moves up, down, and/or side to side with a minimum displacement of 10 centimeters, and the movement is repeated at least twice. | Negative |
| Relaxed tail | The tail moves in sync with the rhythmic motion of the horse's gait. | Positive |
| Tail swishing | The tail moves forcefully from side to side, upward, downward, or in a circular motion. This behaviour is not recorded when it is a response to insects or associated with a forward gait transition. | Negative |
| Moving away | Within a 2-meter radius, the horse's head and neck move away from the client, increasing the distance between them. | Negative |
| Approach human | Within a 2-meter radius, the horse's head and neck move toward the client, reducing the distance between them for at least 3 seconds, with the possibility of the horse initiating physical contact. | Positive |
| Snort | An audible exhalation through the nose lasting a few seconds, during which the mouth and nose muscles remain relaxed. | Positive |
| Blow | A short, sudden, audible exhalation through the nose, accompanied by a closed mouth and tension in the muscles of the mouth and nose. | Negative |
| Tight mouth | The mouth is tightly closed, with visible tension in the jaw muscles and the areas around the nose, lips, and corners of the mouth, sustained for at least 3 seconds. | Negative |
| Yawning | The mouth is held wide open for at least 3 seconds, often revealing the teeth, and occasionally the upper and lower jaws move from side to side. | Negative |
| Licking and chewing | With relaxed mouth, nose, and jaw muscles, the horse exhibits clear licking and chewing behaviour for at least 3 seconds, sometimes with the tongue visible. | Negative |
| Rolling | The horse moves on the ground, rolling from a sternal position to a lateral position, with all four legs lifting off the ground; rolling over the withers is not required. | Negative |
| Defecate | The release of (in)digested feed through the anus. | Negative |
| Self-grooming | Nibbling or manipulating its own skin with the mouth and teeth for at least 3 seconds. | Negative |
| Pawing | A repetitive motion (at least twice) of one front leg, scraping the ground, another surface, or the air in a front-to-back movement. | Negative |
| Snapping and biting | The horse uses its lips, mouth, or teeth to nibble or bite the client, another horse, or an object. | Negative |
| Resting hindleg | One hind leg rests on the ground with the front of the hoof, while the horse's weight is supported by the other three legs, causing the pelvis to dip on the side of the resting hind leg. | Negative |
